# Hot water immersion is associated with higher thermal comfort than dry passive heating for a similar rise in rectal temperature and plasma interleukin-6 concentration

**DOI:** 10.1007/s00421-023-05336-8

**Published:** 2023-10-23

**Authors:** Yunuo Su, Sven. P. Hoekstra, Christof A. Leicht

**Affiliations:** 1 Peter Harrison Centre for Disability Sport, School of Sport, Exercise and Health Sciences, Loughborough University, Loughborough, LE11 3TU UK; 2 Department of Rehabilitation Medicine, University of Texas Health Science Center at San Antonio, San Antonio, USA

**Keywords:** Rectal temperature, Skin temperature, Inflammation, Thermal comfort, Passive heat

## Abstract

**Purpose:**

To compare the perceptual responses and interleukin-6 (IL-6) concentration following rectal temperature-matched dry heat exposure (DH) and hot water immersion (HWI).

**Methods:**

Twelve healthy young adults (BMI 23.5 ± 3.6 kg/m^2^; age: 25.8 ± 5.7 years) underwent 3 trials in randomised order: DH (air temperature 68.9 °C), HWI (water temperature 37.5 °C), and thermoneutral dry exposure (CON, air temperature 27.3 °C). Blood samples to determine IL-6 plasma concentration were collected; basic affect and thermal comfort, rectal and skin temperature (T_skin_) were assessed throughout the intervention.

**Results:**

Rectal temperature (T_rec_) did not differ between DH (end temperature 38.0 ± 0.4 °C) and HWI (37.9 ± 0.2 °C, P = 0.16), but was higher compared with CON (37.0 ± 0.3 °C; P ≤ 0.004). Plasma IL-6 concentration was similar after DH (pre to post: 0.8 ± 0.5 to 1.4 ± 1.5 pg·ml^−1^) and HWI (0.5 ± 0.2 to 0.9 ± 0.6 pg·ml^−1^; P = 0.46), but higher compared with CON (0.6 ± 0.5 to 0.6 ± 0.4 pg·ml^−1^; P = 0.01). At the end of the intervention, basic affect and thermal comfort were most unfavourable during DH (Basic affect; DH: − 0.7 ± 2.9, HWI: 0.8 ± 1.9, CON 1.9 ± 1.9, P ≤ 0.004; Thermal comfort; 2.6 ± 0.8, HWI: 1.4 ± 0.9 and CON: 0.2 ± 0.4; P ≤ 0.004). Mean T_skin_ was highest for DH, followed by HWI, and lowest for CON (DH: 38.5 ± 1.3 °C, HWI: 36.2 ± 0.5 °C, CON: 31.6 ± 0.7 °C, P < 0.001).

**Conclusion:**

The IL-6 response did not differ between DH and HWI when matched for the elevation in T_rec_. However, thermal comfort was lower during DH compared to HWI, which may be related to the higher T_skin_ during DH.

## Introduction

Acute as well as regular, repeated exposure to heat induces a range of physiological adaptations (Ely et al. 2019). For example, a single passive heating session acutely increases arterial blood flow, energy expenditure and the circulating concentration of inflammatory markers (McCarty et al. 2009; Yildirim et al. 2010; Hoekstra et al. 2020; Brunt and Minson 2021). Moreover, the circulating concentration of the pleiotropic cytokine interleukin (IL)-6 has been shown to increase following a single passive heating session of varying durations and temperatures (Laing et al. 2008; Faulkner et al. 2017; Hashizaki et al. 2018; Hoekstra et al. 2018, 2021; Mansfield et al. 2021). Evidence from infusion studies indicates that acute transient elevation of IL-6 concentration stimulates the release of anti-inflammatory cytokines such as IL-10 and IL-1 receptor antagonist; leading to an anti-inflammatory milieu (Steensberg et al. 2003). Based on this notion, it is suggested that the positive long-term health adaptations following repeated exposure to passive heating (i.e. heat therapy) may be partly explained by the acute inflammatory response occurring following each passive heating session and the subsequent reduction in the concentration of basal pro-inflammatory markers such as tumour necrosis factor-α and C-reactive protein (Petersen and Pedersen 2005; Oyama et al. 2013; Leicht et al. 2015; Ely et al. 2017; Simmons et al. 2017; Hoekstra et al. 2018). Furthermore, blocking IL-6 signalling through tocilizumab abolishes the positive effects of exercise training on adipose tissue mass; suggesting an additional role for repeated transient elevations in IL-6 concentration in lipid metabolism. Thus, in line with the concept of hormesis (Martin et al. 2010), repeated transient elevations of IL-6 concentration appear beneficial for cardiometabolic health, and can be instigated by heat therapy.

Heat therapy is accessible and low-cost, while it has minimal side-effects compared to pharmaceutical agents (Simkin and Bolding 2004). Common modes of heat exposure include dry heat (DH) exposure (e.g., Finnish sauna, far infrared sauna, Waon therapy) and hot water immersion (HWI) (Brunt and Minson 2021). In all modes, passive heat stress raises skin (T_skin_) and rectal temperature (T_rec_), and activates the autonomic nervous system, increasing heart rate (HR), respiration and sweat rate (Kukkonen-Harjula et al. 1989; Jezova et al. 2007; Kikuchi et al. 2013). The specific mode of heating influences the temperature to which the skin is directly exposed. The temperature in a sauna (~ 80–100 °C) (Helamaa and Aikas 1988), exposing the body to DH, differs substantially to the water temperature in typical HWI protocols (~ 38–42 °C) (Cheung and Sleivert 2004; Goto et al. 2011; Hoekstra et al. 2018). As the heat transfer is 24 times higher in water than in air (Nadel 1977), substantially lower water temperatures can achieve a similar increase in T_rec_ (Brunt and Minson 2021). However, the higher environmental temperature during DH is likely to induce a higher rise in T_skin_ when compared with HWI (Campbell et al. 2022). A difference in T_skin_ between DH and HWI may affect peripheral muscle temperature (Chiesa et al. 2015), which may hence affect inflammatory responses, as a high skeletal muscle temperature can stimulate muscle IL-6 release (Welc et al. 2012). Although an acute increase in circulating IL-6 concentration has been observed for both DH and HWI (Kaldur et al. 2016; Hoekstra et al. 2018), it is unknown how the inflammatory response compares between both heating modes.

Given that T_skin_ is a major determinant of thermal comfort (Frank et al. 1999), perceptual responses may differ between DH and HWI. Furthermore, HWI may cause skin maceration and skin softening (An et al. 2019), which may also affect perception. On the other hand, sweaty clothing in DH may also cause discomfort (Jiang and Wang 2020). These factors are highly relevant when prescribing heat therapy regimens, as individuals are more likely to repeat behaviour they find comfortable and from which they derive pleasure (Jung et al. 2014; Hoekstra et al. 2018). At the same time, the health effects of heat therapy appear to be dose-dependent (Zaccardi et al. 2017; Kunutsor et al. 2018), exemplified by a 40% lower risk of all-cause mortality in frequent compared to infrequent sauna users (Laukkanen et al. 2015). Thus, insight into factors promoting adherence to heat therapy are important for the success of future long-term interventions.

The aim of this study was therefore to compare the inflammatory and perceptual responses to rectal temperature-matched DH and HWI. It was hypothesised that (1) DH induces a larger rise in T_skin_ than HWI, (2) due to the higher T_skin_ in DH, the acute elevation of IL-6 is higher in DH compared with HWI and (3) perceptions during HWI are more positive than in DH.

## Methods

### Participants

Participants were 12 healthy young adults (3 females, 9 males; age: 25.8 ± 5.7 years; height: 177.2 ± 9.3 cm; weight: 74.3 ± 14.2 kg; self-reported structured exercise: 4.0 ± 3.1 h/week). All participants in the study filled out a standard health questionnaire used throughout the Department at Loughborough University to ensure no contraindications for heat therapy were present. Further exclusion criteria were smoking, the use of anti-inflammatory medication and engaging in structured exercise for more than 8 h/week. In line with the Declaration of Helsinki, participants gave written informed consent after being informed of the study procedures, approved by Loughborough University’s ethics committee (code: 1308).

### Experimental design

**Fig. 1:**
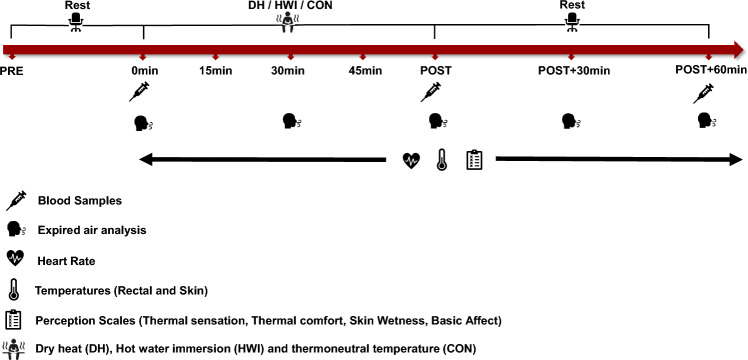
Experimental design and timing of data collection

Participants visited the laboratory to undergo three experimental conditions in a randomised order: DH exposure, HWI and a control condition (CON) (Fig. 1). During DH, participants maintained a supine position in the Cocoon POD (Wellness USA, Minneapolis, Minnesota, USA). The entire body except the head was exposed to DH. The Cocoon POD setting used was ‘high-hyper’ (the highest temperature setting), resulting in an air temperature of 68.9 ± 3.2 °C and 7.8 ± 2.7% humidity. For HWI, the water temperature was set at 37.5 °C and maintained manually. Participants maintained a seating position and were immersed in water up to the neck. The water temperature was chosen with the aim to match the T_rec_ rise observed in the Cocoon POD, as determined during pilot testing. During CON, participants rested in the Cocoon POD at a thermoneutral temperature (27.3 ± 1.4 °C and 39.4 ± 4.8% humidity). The temperature and humidity in the Cocoon POD were measured using an I-button (Homechip Ltd, Milton Keynes, UK) attached to the roof of the device, at the level of the participants’ chest. All visits began between 11 am and 1 pm to control for potential circadian rhythm variation in any of the outcome measures. Visits were separated by a minimum of 48 h and were completed within 60 days. Participants were asked to avoid exercise, caffeine, and alcohol intake the day before the laboratory visit. In addition, they kept a 24-h dietary record before the first visit and replicated that diet for subsequent visits.

At the start of every session, the following devices were applied and worn throughout the entire trial: (1) a rectal probe (YSI Precision ™ 4000A Thermometer, Ohio, USA) inserted ~ 10 cm past the anal sphincter for the measurement of T_rec_, (2) six T_skin_ sensors (I-buttons, Homechip Ltd, Milton Keynes, UK) placed on the skin (forehead, cheek, chest, arm, thigh, calf) to measure T_skin_, (3) a Polar heart rate monitor (Polar, Kempele, Finland) to monitor HR. Body mean T_skin_ was calculated as a weighed mean of the arm, chest, thigh and calf (Ramanathan 1964):

$${\text{Mean T}}_{{{\text{skin}}}} = \, 0.{3}*{\text{chest T}}_{{{\text{skin}}}} + \, 0.{3}*{\text{arm T}}_{{{\text{skin}}}} + 0.{2}*{\text{thigh T}}_{{{\text{skin}}}} + \, 0.{2}*{\text{calf T}}_{{{\text{skin}}}}$$

Aside from the absolute values, the rate of increase in mean T_skin_ was calculated from baseline to peak T_skin_. During the intervention, expired air was collected every 30 min and perceptual responses were reported every 15 min. Expired air was collected in Douglas bags over a three-minute period. Expired air was analysed using a Servomex 1440 gas analyser (Servomex Ltd, Crowborough, UK) to obtain oxygen uptake and carbon dioxide production rates to determine energy expenditure (de Weir 1949). The following perceptual responses were reported using visual analogue scales: thermal sensation (1 very cold–9 very hot) (Epstein and Moran 2006), thermal comfort (0 comfortable to + 4 very uncomfortable) (Gagge and Nishi 1976), skin wetness perception (face and other parts of the body; − 3 very dry to + 3 very wet) (Filingeri et al. 2015), basic affect (i.e., ‘how do you feel at this moment in time?’; − 5 very bad to + 5 very good) (Hardy and Rejeski 1989).

Following the measurement of height and nude body mass, participants rested in a seated position for 30 min. After that, physiological and temperature measurements were obtained, while participants also reported their thermal sensation, thermal comfort, basic affect and skin wetness perception. The participants then entered the Cocoon POD (DH) or water (HWI) for 60 min with measurements taken every 15 min. During all visits, water intake was allowed ad libitum. At the end of 60 min, nude body mass was recorded again for the estimation of sweat loss, taking any water consumed into account. Participants then rested in a seated position for a further 60 min. They were allowed to perform non-strenuous tasks such as reading or working on a laptop. Further measurements were obtained 30 and 60 min after the session. At the end of each visit, participants were asked to rate their enjoyment of the intervention on a 20 cm visual analogue scale (0—not pleasant to 20—very pleasant). After completing all three trials, participants were asked to select their favourite trial, provide a fondness rating (on a scale from 1 least liked to 9 most liked) for each session, and explain why they preferred the chosen session.

### Biochemical analysis

Blood was collected in tri-potassium EDTA monovettes, which were spun at 3500 rpm and 4 °C for 10 min. The plasma was divided into aliquots and stored at − 80 °C until batch analysis. Plasma IL-6 was measured using a high sensitivity enzyme-linked immunosorbent assay (ELISA) kit according to the manufacturer’s instructions (R&D Systems, Abingdon, UK), using a microplate reader (Varioskan Flash, ThermoScientific, Waltham, US). The intra-assay coefficient of variation was 6.4%. Haematocrit, determined in duplicate using a microcentrifuge, and haemoglobin concentration, determined by a Yumizen H500 automated analyser (Horiba Medical, Montpellier, France) were used to correct IL-6 concentrations for changes in plasma volume (Dill and Costill 1974).

### Statistical analysis

The statistical software SPSS 25.0 (Chicago, IL) was used for all statistical analyses. Results are expressed as mean and standard deviation. Data were checked for normality using the Shapiro–Wilk test and log-transformed when non-normality was detected. A repeated measures analysis of variance (ANOVA) was then performed for IL-6 and body temperature measures, followed by post-hoc pairwise comparisons in case of a statistically significant main effect. In cases where sphericity assumptions were violated, Greenhouse–Geisser corrections were applied. The effect size (ES; Cohen's d) was calculated for IL-6 outcomes using the difference between Pre- and Post-change scores in each condition (0.20–0.50 small, 0.50–0.80 moderate, > 0.80 large effect (Cohen 1992). Changes in perceptual responses and energy expenditure were analysed non-parametrically. First, the Friedman test was used to test for an effect of time in each condition, whereafter Wilcoxon signed-rank tests were performed to compare individual time points between conditions. Finally, correlations were computed using Spearman’s r, investigating bivariate relationships between temperature and thermal perception variables, as well as temperature and plasma markers measured at the end of the intervention. P values are reported without a Bonferroni correction (Perneger 1998). For all analyses, the statistical significance threshold was set at P < 0.05.

## Results

### Body temperature

**Fig. 2:**
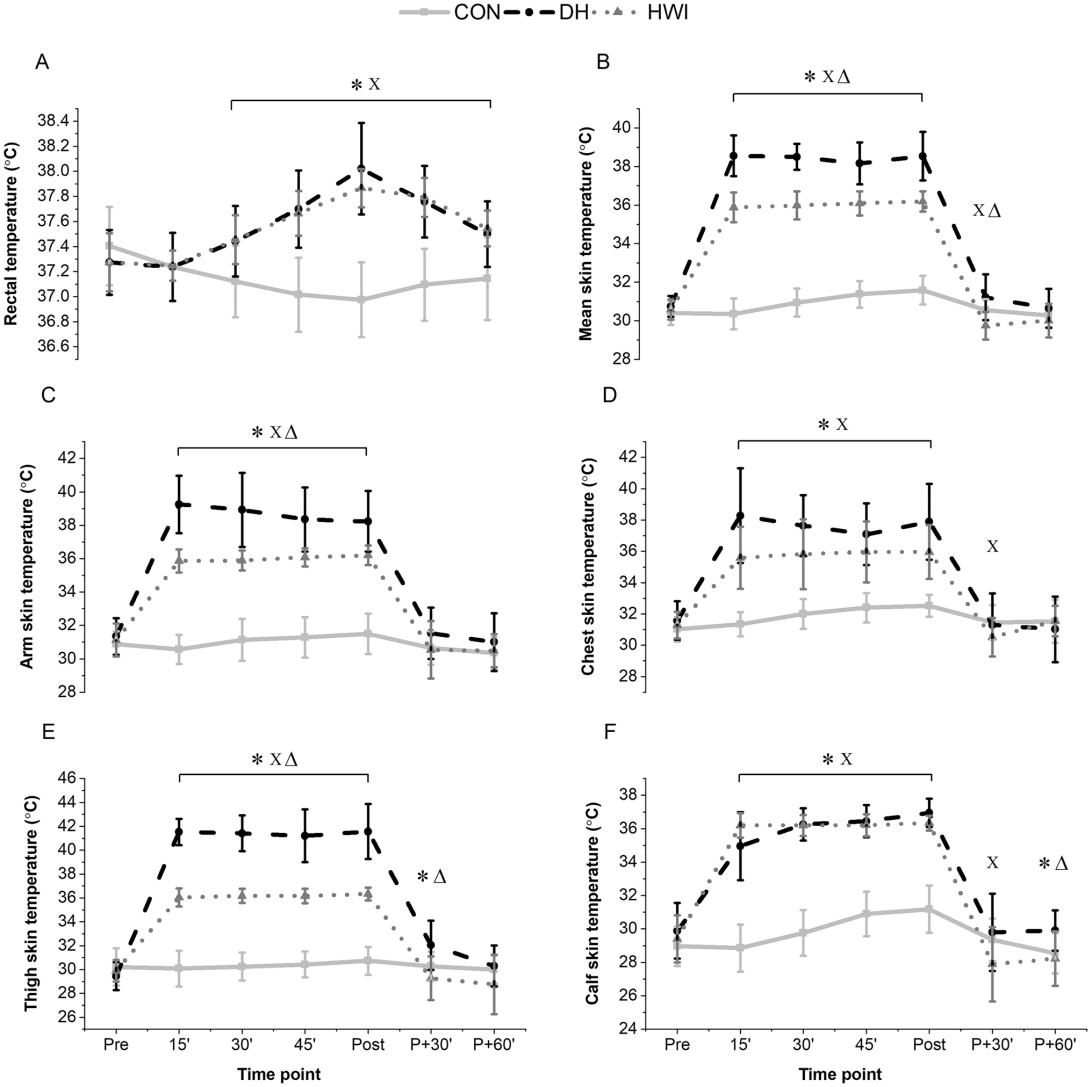
Acute changes in rectal temperature (**A**), mean skin temperature (**B**) and skin temperatures for arm (**C**), chest (**D**), thigh (**E**) and calf (**F**) in response to Thermoneutral temperature (CON), dry heat (DH) and hot water immersion (HWI). Data reported as mean and standard deviation. Significant difference between *DH and CON, ^Χ^HWI and CON and ^Δ^DH and HWI (P < 0.05), determined via ANOVA

A main effect of time (F_(6,66)_ = 41.0, P < 0.001), condition (F_(2,22)_ = 13.0, P < 0.001) and a time by condition interaction (F_(12,132)_ = 41.0, P < 0.001) indicated a differential T_rec_ increase between conditions. The peak T_rec_ did not differ between DH (38.0 ± 0.4 °C) and HWI (37.9 ± 0.2 °C; F_(2,22)_ = 45.9, P = 0.16; Fig. 2A) but was higher in both conditions compared with CON (37.0 ± 0.3 °C; F_(2,22)_ = 45.9, P ≤ 0.004).

A main effect of time (F_(6,66)_ = 585.3, P < 0.001), condition (F_(2,22)_ = 271.6, P < 0.001), and time by condition interaction (F_(12,132)_ = 108.5, P < 0.001) was found for mean T_skin_. Mean T_skin_ was higher in DH (38.5 ± 1.2 °C), compared with HWI (36.1 ± 0.6 °C); and was higher throughout both these conditions compared with CON (31.6 ± 0.7 °C; F_(2,22)_  ≥ 208.8, P < 0.001, Fig. 2B). The rate of rise in mean T_skin_ during the first 15 min was 0.53 °C/min in DH and 0.35 °C/min in HWI.

Arm and thigh T_skin_ were higher for DH than for HWI across the entire intervention (F_(2,22)_ ≥ 68.2, P ≤ 0.013, Fig. 2C, E). Chest and calf T_skin_ during DH and HWI were higher than during CON (F_(2,22)_ ≥ 22.4, P < 0.001, Fig. 2D, F).

### Inflammatory response

**Fig. 3:**
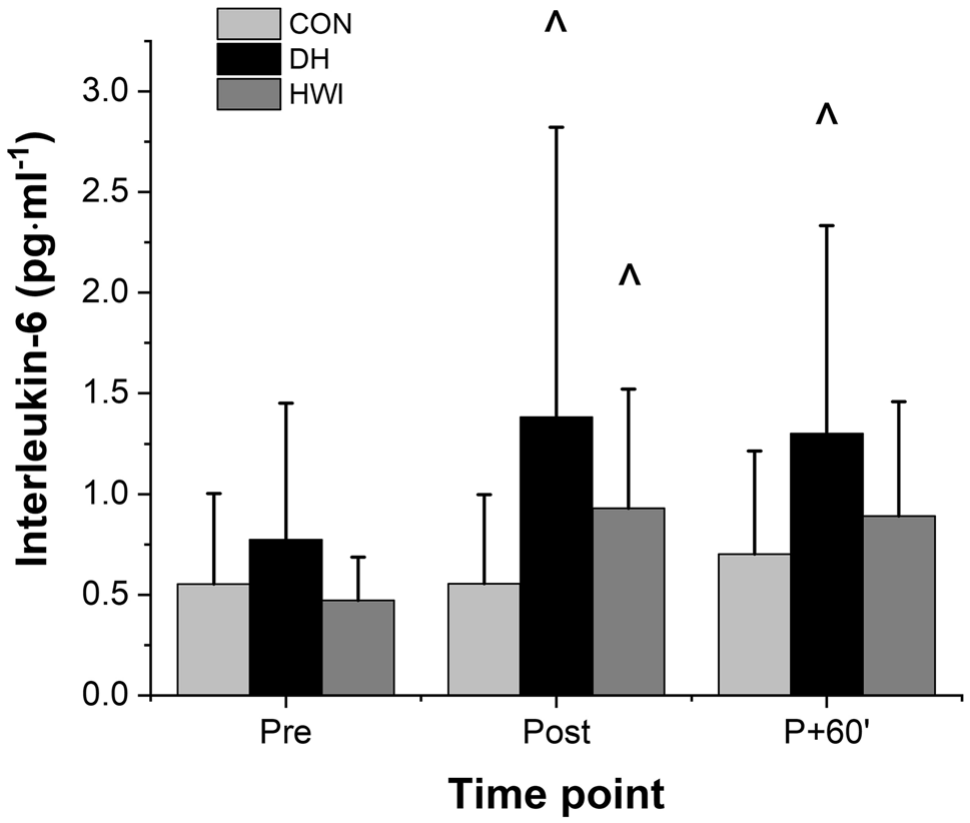
Acute changes in plasma IL-6 concentration in response to Thermoneutral temperature (CON), Dry heat (DH) and Hot water immersion (HWI). Data reported as mean and standard deviation. ^Significantly higher than CON (P < 0.05), determined via ANOVA

A main effect of time (F_(2,22)_ = 28.4, P < 0.001), condition (F_(2,22)_ = 4.1, P = 0.031), and time by condition interaction (F_(4,44)_ = 6.3, P = 0.007) was found for plasma IL-6 concentration (Fig. 3). Immediately post heating, plasma concentrations of IL-6 were elevated in DH (F_(2,22)_ = 7.1, P = 0.005) and HWI (F_(2,22)_ = 7.1, P = 0.011) compared with CON; however, DH and HWI did not differ from each other (F_(2,22)_ = 7.1, P = 0.458). At post 60 min, IL-6 concentrations were higher for DH than CON (F_(2,22)_ = 4.4, P = 0.019), but HWI did not differ from CON (F_(2,22)_ = 4.4, P = 0.067). There was no difference in plasma IL-6 concentrations between Pre and Post in CON (P = 0.943; d = 0.079), but plasma IL-6 concentrations significantly increased from pre to post in DH (P = 0.045; d = 0.925) and HWI (P = 0.003; d = 0.412). Plasma IL-6 concentrations were different between Pre and Post 60 min in all conditions; CON (P = 0.046; d = 0.228), DH (P = 0.011; d = 0.598) and HWI (P = 0.009; d = 0.456).

### Perceptual responses

During both DH and HWI, basic affect, thermal sensation, thermal comfort, face wetness and body wetness changed over time (P ≤ 0.002), which was not the case for CON (P > 0.130, Table 1). From fifteen min into the session, basic affect scores were more unfavourable for DH when compared with CON (P ≤ 0.041), whilst HWI and CON did not differ at 15 min (P = 0.380) and at 45 min (P = 0.060) of the intervention. From 30 min, basic affect was more unfavourable during DH (P ≤ 0.007). During DH, basic affect was in the range of − 1 (“fairly bad”), whilst it was in the range of 1–2 (“fairly good” to “good”) during HWI and CON.

**Table 1:** Perceptions in response to dry heat and hot water immersion

Parameter	Conditions	Time point						
		Pre	15′	30′	45′	Post	Post + 30′	Post + 60′
Basic affect (− 5 to + 5)	CON	2.3 ± 1.4	2.2 ± 1.6	2.1 ± 1.4	2.3 ± 1.7	1.9 ± 1.9	2.1 ± 1.6	1.9 ± 1.6
	DH^**†**^	2.2 ± 1.3	1.4 ± 1.6*	0.6 ± 1.7*^Δ^	0.2 ± 2.1*^Δ^	− 0.7 ± 2.9*^Δ^	1.8 ± 1.9	1.9 ± 1.8
	HWI^**†**^	1.9 ± 1.7	1.8 ± 1.3	**1.1 ± 1.5**^**Χ**^	1.3 ± 1.5	**0.8 ± 1.9**^**Χ**^	1.8 ± 1.7	1.8 ± 1.7
Thermal sensation (1 to 9)	CON	4.8 ± 0.6	4.8 ± 0.9	4.7 ± 0.8	4.6 ± 0.8	4.7 ± 0.7	4.8 ± 0.6	4.4 ± 0.9
	DH^**†**^	4.8 ± 0.6	7.2 ± 0.6*^Δ^	7.7 ± 0.5*^Δ^	8.2 ± 0.6*^Δ^	8.4 ± 0.5*^Δ^	4.5 ± 0.5	4.3 ± 1.0
	HWI^**†**^	4.6 ± 0.7	**6.3 ± 0.6**^**Χ**^	**6.9 ± 0.8**^**Χ**^	**7.1 ± 0.7**^**Χ**^	**7.1 ± 0.7**^**Χ**^	4.5 ± 0.5	4.3 ± 0.6
Thermal comfort (0 to 4)	CON	0.3 ± 0.6	0.2 ± 0.4	0.1 ± 0.3	0.1 ± 0.3	0.2 ± 0.4	0.2 ± 0.6	0.3 ± 0.6
	DH^**†**^	0.1 ± 0.3	1.0 ± 0.6*	1.7 ± 0.8*^Δ^	1.8 ± 0.9*^Δ^	2.6 ± 0.8*^Δ^	0.3 ± 0.5	0.3 ± 0.5
	HWI^**†**^	0.3 ± 0.6	0.5 ± 0.7	**1.1 ± 0.7**^**Χ**^	**0.9 ± 0.7**^**Χ**^	**1.4 ± 0.9**^**Χ**^	0.2 ± 0.4	0.3 ± 0.5
Face wetness (-3 to + 3)	CON	− 1.1 ± 1.1	− 0.8 ± 1.1	− 0.8 ± 1.1	− 0.9 ± 1.2	− 0.8 ± 1.1	− 0.8 ± 1.1	− 0.8 ± 1.1
	DH^**†**^	− 0.8 ± 1.1	0.7 ± 1.1*	1.8 ± 1.0*^Δ^	2.4 ± 0.8*	2.6 ± 0.7*^Δ^	− 0.2 ± 1.1	− 0.5 ± 0.8
	HWI^**†**^	− 0.8 ± 1.2	**0.0 ± 1.3**^**Χ**^	**1.4 ± 1.0**^**Χ**^	**1.7 ± 1.6**^**Χ**^	**1.8 ± 1.3**^**Χ**^	− 0.4 ± 1.2	− 0.6 ± 0.9
Body wetness (− 3 to + 3)	CON	− 0.9 ± 1.1	− 0.8 ± 1.1	− 0.8 ± 1.0	− 0.8 ± 1.2	− 0.8 ± 1.1	− 0.6 ± 1.2	− 0.8 ± 1.1
	DH^**†**^	− **0.1 ± 1.2**^**Δ**^	1.0 ± 0.6*^Δ^	2.0 ± 0.9*^Δ^	2.7 ± 0.5*	2.7 ± 0.5*	0.7 ± 1.0*	− 0.3 ± 1.0
	HWI^**†**^	− 0.9 ± 1.2	**2.4 ± 1.0**^**Χ**^	**2.8 ± 0.5**^**Χ**^	**2.8 ± 0.5**^**Χ**^	**2.8 ± 0.5**^**Χ**^	**0.4 ± 1.4**^**Χ**^	**0.1 ± 1.2**^**Χ**^

Data reported as mean ± SD. Significant differences are highlighted in bold. *CON* Thermoneutral temperature, *DH* dry heat, *HWI* hot water immersion

^**†**^Effect of time (*P* < 0.05), as analysed using the Friedman test. Significant difference between *DH and CON, ^Χ^HWI and CON and ^Δ^DH and HWI (*P* < 0.05), as analysed using Wilcoxon signed-rank test

Thermal sensation differed between all conditions at all time points of the intervention; the most unfavourable scores were reported during DH, followed by HWI and CON (P ≤ 0.024, Table 1). Thermal comfort scores were most unfavourable for DH when compared with CON (P = 0.004), whilst HWI and CON did not differ at 15 min (P = 0.157). After 30 min, thermal comfort differed between all conditions (P ≤ 0.035) and was in the range of + 2 (“uncomfortable”) for DH; in the range of + 1 (“slightly uncomfortable”) for HWI and in the range of 0 (“comfortable”) for CON. None of the perceptual responses differed in the recovery period (P > 0.10 for all comparisons).

Perceived face wetness for DH and HWI were both higher than for CON at all time points of the intervention (P ≤ 0.011); DH face wetness was greater at 30 and 60 min than HWI (P ≤ 0.025). Perceived body wetness for DH was higher than HWI at baseline (P = 0.023). During DH and HWI, body wetness scores were greater throughout the intervention period, including 30 min after the intervention than CON, and remained higher for HWI after 60 min (P ≤ 0.036).

Enjoyment scores were lower for DH (8.7 ± 5.2) than for HWI (13.5 ± 2.9; P = 0.013) and CON (12.6 ± 2.9; P = 0.021), with no difference between HWI and CON (P = 0.126). Fondness scores﻿ were higher for HWI (6.3 ± 1.8) than for CON (4.5 ± 2.6; P = 0.038). There was no difference in fondness for DH (4.7 ± 2.3) compared with CON (P = 0.905) and HWI (P = 0.097).

HWI was selected as the favourite condition by N = 7, DH by N = 3, and CON by N = 2. Qualitative feedback following the intervention to support this choice included: “DH is slightly uncomfortable”; “DH is intensely hot”; “DH is easier than HWI”; “More pleasant/relaxing in the water”; “HWI more comfortable than DH”; “Not too hot on the skin in HWI”.

### Cardiovascular and sweat response

A main effect of time (P < 0.001), condition (P < 0.001) and a time by condition interaction (P < 0.001) indicated a differential HR increase between conditions. Heart rate was higher for DH and HWI when compared with CON during the experimental period, and following that, HR for HWI remained higher than CON at one hour post (P < 0.001).

Energy expenditure for DH and HWI changed over time (P ≤ 0.001), with values in DH higher than CON at 60 min (P = 0.028). Sweat loss was higher during DH and HWI when compared with CON (P ≤ 0.001). In addition, sweat loss was higher following DH compared to HWI (P < 0.001, Table 2).

**Table 2:** Physiological responses to the experimental conditions

Parameter	Conditions	Time point				
		Pre	30′	Post	Post + 30′	Post + 60′
Heart rate (bpm)	CON	66 ± 12	60 ± 11	61 ± 10	64 ± 9	61 ± 10
	DH^**†**^	61 ± 12*	80 ± 10*	91 ± 9*	69 ± 13	65 ± 12
	HWI^**†**^	64 ± 9*	85 ± 10*	90 ± 10*	68 ± 12*	66 ± 12*
Energy expenditure (kcal/min)	CON	79 ± 21	73 ± 21	72 ± 24	71 ± 18	71 ± 19
	DH^**†**^	83 ± 28	86 ± 31	92 ± 32*	82 ± 29	67 ± 25
	HWI^**†**^	68 ± 29	87 ± 33	86 ± 29	76 ± 28	76 ± 28
Sweat loss (ml)	CON	N/A	N/A	90 ± 52	N/A	N/A
	DH	N/A	N/A	815 ± 359*^Δ^	N/A	N/A
	HWI	N/A	N/A	566 ± 382*^Χ^	N/A	N/A

Data reported as mean ± SD. Significant differences are highlighted in bold. *CON* Thermoneutral temperature, *DH* dry heat, *HWI* hot water immersion

^**†**^Effect of time (*P* < 0.05). Significantly different from *CON, ^Χ^DH and ^Δ^HWI (*P* < 0.05). Heart rate and sweat loss were analysed using ANOVA; energy expenditure using the Wilcoxon signed-rank test

### Bivariate relationships

In DH, thermal sensation correlated with arm T_skin_ (r = − 0.61, P = 0.034), and facial wetness was significantly correlated with body wetness (r = 0.65, P = 0.023). In HWI, basic affect was correlated with mean T_skin_ (r = 0.65, P = 0.024), and thermal sensation was correlated with calf T_skin_ (r = 0.71, P = 0.010). There was no relationship between face wetness and body wetness in HWI (r = 0.41, P = 0.190). In addition, IL-6 plasma concentrations did not correlate significantly with T_rec_ (DH: P = 0.505, HWI: P = 0.649, CON: P = 0.412) and mean T_skin_ (DH: P = 0.991, HWI: P = 0.457, CON: P = 0.233) in any of the conditions.

## Discussion

This study investigated the acute effects of T_rec_-matched DH and HWI on inflammatory as well as perceptual responses. The main results were: (1) mean T_skin_ during DH was higher when compared with HWI; (2) the plasma IL-6 response did not differ between DH and HWI; (3) basic affect, thermal comfort, and thermal sensation during HWI were more positive when compared with DH.

### Inflammatory response

As DH and HWI showed the same IL-6 and T_rec_ increase but differed substantially in the T_skin_ increase, the results of the present study may therefore imply that T_rec_ is a more important driver to elevate IL-6 plasma concentration than T_skin_ . The T_rec_ increase in the present study was relatively modest (~ 0.7 °C), which explains the relatively modest increase in IL-6 plasma concentration (~ twofold). Previous passive heating studies employed more aggressive heating protocols (T_rec_ increase ~ 1–2 °C) and observed ~ two- to threefold increases in IL-6 plasma concentrations (Leicht et al. 2015; Faulkner et al. 2017; Hoekstra et al. 2018). This is in line with the suggested dose–response relationship between heat exposure, associated elevated T_rec_ and the acute IL-6 response (Brunt et al. 2018; Hoekstra et al. 2020).

In the present study, the measure for peripheral temperature, mean T_skin_, was higher in DH than in HWI. T_skin_ has been suggested to be related to sympathetic nervous system activity (Andrasik and Rime 2011), as the latter plays an important role in thermoregulation by regulating skin blood flow and perspiration (Collins 2013). As sympathetic activation is associated with the IL-6 response (Okamoto et al. 2015), the higher T_skin_ during DH may have been expected to augment the IL-6 response via greater sympathetic activation. However, HR, another indicator of sympathetic activity, was not different between DH and HWI, suggesting that sympathetic activation may have been similar between these trials.

In addition to sympathetic activation, the acute heat stress-induced inflammatory response may be influenced by peripheral tissue temperature (Kaldur et al. 2016). For instance, an ex vivo study showed increased IL-6 mRNA and protein expression during skeletal muscle heating (Welc et al. 2012). Further, Hoekstra et al., (2021) showed that the IL-6 response during passive heating was not blunted when the T_rec_ increase during heating of the legs was blunted by upper body cooling. Therefore, it may be that a large increase in T_rec_ is not required for a whole-body IL-6 response to occur as long as skeletal muscle, a main producer of IL-6 (Welc et al. 2012), is heated. Despite this reported influence of peripheral (muscle and/or skin) temperature on the IL-6 response, DH, which led to a higher rise in T_skin_, did not lead to higher IL-6 concentrations in the current study. Here we must note that it is also possible that the observed differences in T_skin_ did not translate to divergent muscle temperatures (not directly measured in this study) between DH and HWI. A previous DH study showed that thigh T_skin_ was higher than the lateral femoral muscle temperature (Raccuglia et al. 2016). Conversely, a HWI study using 42 °C water reported higher lateral femoral muscle temperatures than T_skin_ (Rodrigues et al. 2020). Thus, this discrepancy between muscle temperature and T_skin_ for different modes of heating does not allow firm predictions of muscle temperature in the present study, which would require direct measures of muscle temperature in follow-up studies.

Elevated basal concentrations of IL-6 have repeatedly been associated with vascular dysfunction and impaired metabolic health (de Winther et al. 2005). Repeated passive heating can exert a downregulating effect on basal IL-6 concentration. For example, a study involving two weeks of hot spring bathing revealed a reduction in circulating IL-6 concentration among patients diagnosed with chronic heart failure (Oyama et al. 2013), highlighting the potential therapeutic impact of heat stress on modulating inflammatory responses. The current study indicates that even modest heat stress can induce an acute elevation in IL-6 concentration. This is in line with other studies, as heating of peripheral tissue resulting in a moderate rise in T_rec_ (~ 0.6 °C) has demonstrated the potential to acutely elevate plasma IL-6 levels (Kaldur et al. 2016). Importantly, the comparable increase in IL-6 concentration observed following HWI and DH implies that individuals may make their selection of heating modality based on their personal preference or practical considerations.

### Perceptual responses

It has been suggested that thermal sensation is mainly affected by T_skin_, while thermal comfort is affected by both T_skin_ and T_rec_ (Kato et al. 2001). In the present study, T_skin_ during DH was higher than during HWI, whilst the increase in T_rec_ was similar between these conditions. In addition, the thermal sensation was greater in DH compared to HWI, and thermal comfort was more negative in DH than in HWI after 30 min of passive heating, indicating that DH was less tolerable than HWI. Our data hence support the mechanistic role of T_skin_ in thermal perceptions (Chatonnet and Cabanac 1965) and support the suggestion that the higher T_skin_ in DH may be the reason why DH is perceived as hotter and more uncomfortable than T_rec_-matched HWI. This is supported by correlational analyses from the current study, where T_skin_ outcomes were associated with thermal sensation and basic affect in both DH and HWI, respectively. Our findings are further corroborated by a previous study documenting a majority of participants not being able to complete 60 min of sauna exposure (55 °C, 54% relative humidity) due to discomfort, whereas all tolerated 60 min of water immersion (40 °C) resulting in similar T_rec_, but lower T_skin_ when compared with sauna exposure (Campbell et al. 2022). Of note, while T_skin_ during DH and HWI most likely reflects the exposure of the skin to the different thermal stimulus (i.e., water of ~ 37.5 °C and air of ~ 70 °C), it is important to acknowledge the potential direct impact of the water and air temperatures on the T_skin_ readings as a limitation.

Face wetness and body wetness perception were affected differently by DH and HWI. Face wetness perception was more pronounced in DH than in HWI, which may be related to the higher sweat rate during DH. Importantly, the higher face wetness during DH may also have affected the thermal comfort in this condition, as a correlation between these variables has been identified previously (Fukazawa and Havenith 2009). In DH, although dry air allows the body to dissipate heat through sweat evaporation, it must be noted that the Cocoon POD is a closed, capsule-like construction, limiting air flow to the skin and, therefore, evaporative heat loss. This may impair the evaporation efficiency of sweat, which increases the sensation of heat (Lee et al. 2011).

HWI was rated highest in both enjoyment and fondness, as well as being most often selected favourite condition. As indicated by the Hedonic theory, behaviours are more likely to be repeated if they are associated with pleasure (Ekkekakis et al. 2008). Taking this into account, our findings suggest that HWI could potentially be a superior method to DH when prescribing long-term heat therapy. However, it is important to acknowledge the relatively small sample size in this study and the fact that HWI was chosen as favourite by 58% of participants rather than close to anonymously. Further research with a larger and more diverse group of participants would be beneficial to gain a more comprehensive understanding of the preferences and experiences related to these conditions.

### Cardiovascular and sweat response

Heart rate was increased during both DH and HWI to ~ 90 beats per minute, which is common during sauna therapy, even though a maximum HR of up to 120–150 beats per minute has also been reported (Laukkanen et al. 2018). A difference between DH and HWI is the hydrostatic pressure exerted on the body (Arborelius et al. 1972). The hydrostatic pressure during HWI did not appear to affect HR in this study, consistent with a study conducted post-exercise with DH or HWI, that reported no difference in HR between the two heating modes (Ashworth et al. 2023). A lower HR during HWI might have been expected, as HWI can cause central blood volume expansion, increasing stroke volume whilst decreasing HR compared with non-immersion (Wilcock et al. 2006). It therefore appears that the effect of the heat stress on HR was more dominant than the effect of hydrostatic pressure. Finally, a previous study shows T_skin_ to be an important determinant of the sweat response when T_rec_ is fixed (Gagnon and Crandall 2018). This aligns with our findings, where T_skin_ and sweat loss were greater for DH than for HWI while matched for the rise in T_rec_.

## Conclusion

This study showed that when matched for the rise in T_rec_, DH and HWI result in a similar IL-6 response. At the same time, perceptual responses were more positive during HWI when compared with DH, which may be related to the lower mean T_skin_ during HWI. Reducing T_skin_ while ensuring an increase in T_rec_ may thus enhance the tolerability of passive heating without impacting the IL-6 response.

## Data Availability

Data will be made available upon request.

## Abbreviations

ANOVA: Analysis of variance
CON: Control condition, thermoneutral dry exposure
DH: Dry heat
HR: Heart rate
HWI: Hot water immersion
IL-6: Interleukin-6
T_rec_: Rectal temperature
T_skin_: Skin temperature
